# Is Anyone Listening? Variation in PSA Screening among Providers for Men 75+ before and after United States Preventive Services Task Force Recommendations against It: A Retrospective Cohort Study

**DOI:** 10.1371/journal.pone.0107352

**Published:** 2014-09-10

**Authors:** James S. Goodwin, Elizabeth Jaramillo, Liu Yang, Yong-Fang Kuo, Alai Tan

**Affiliations:** 1 Sealy Center on Aging, University of Texas Medical Branch, Galveston, Texas, United States of America; 2 The Department of Internal Medicine, University of Texas Medical Branch, Galveston, Texas, United States of America; 3 The Department of Preventive Medicine and Community Health, University of Texas Medical Branch, Galveston, Texas, United States of America; Carolina Urologic Research Center, United States of America

## Abstract

**Background:**

In 2008, the United States Preventive Services Task Force recommended against prostate specific antigen (PSA) testing for cancer screening in men age 75+.

**Purpose:**

To assess PSA screening by primary care physicians (PCPs) before and after recommendations.

**Methods:**

In 2013, this retrospective cohort study analyzed PCPs in Texas with 20+ male patients aged 75+ in both 2007 and 2010, with Parts A and B Medicare. The main outcome was percent of PCP’s male patients 75+ who received PSA testing ordered by the PCP in 2007 and 2010, with no recent symptoms suggestive of prostate cancer.

**Results:**

In both 2007 and 2010, 1,083 PCPs cared for at least 20 men aged 75 or older. The rate of PSA screening ordered by PCPs was 33.2% in 2007 and 30.6% in 2010. In multilevel analyses controlling for patient characteristics, the variation in PSA screening attributable to the PCP (intraclass correlation coefficient) increased from 23% in 2007 to 26% in 2010, p<0.001. Men with PCPs older than age 60 had 9% lower odds (95% CI, 1–17%) in 2010 compared to 2007 of receiving a PSA test, vs. a 4% increase (95% CI, 4% decrease to 12% increase) in men with PCPs aged 50 or younger. Patients with Board Certified PCPs had a 12% lower odds (95% CI, 8% to 16%) from 2007 to 2010, vs. 2% increase (95% CI 11% decrease to 18% increase) in men with PCPs without board certification.

**Conclusions:**

The USPSTF recommendation did not increase consensus among PCPs regarding PSA screening of older men.

## Introduction

The introduction of routine prostate specific antigen (PSA) testing was associated with increases in the number of men diagnosed with and treated for prostate cancer in the U.S. [1] For example, for every 100,000 men aged 66–74 receiving PSA testing in the US in 2007, an additional 4,894 men underwent prostate cancer biopsy, and 1,597 were treated [2].

Prostate cancer is especially problematic in older men, who may benefit little from its diagnosis and treatment. Because of this, in 2008 the US Preventive Services Task Force (USPSTF) specifically recommended against PSA screening in men aged 75 and older. [3] Other organizations, including the American Cancer Society and the American Urological Association, also recommend against routine PSA testing in men over 70 [4] or in men with life expectancy less than 10–15 years. [5], [6] Nevertheless, high rates of PSA testing continue, with relatively modest decreases in PSA testing rates in men over age 75. [7]–[12] One complicating issues is distrust among physicians and patients of guidelines that recommend less care [13], [14].

Physician recommendation is a major driver of testing, along with patient knowledge and preferences. [15], [16] A previous study used Medicare data to describe substantial variation in PSA screening rates among different primary care physicians (PCPs). [10] Variation among providers is thought to reflect lack of evidence, and variation should decrease as underlying evidence grows and provider consensus increases. [17], [18] This report compares PSA screening rates by Texas PCPs for their male patients aged 75 years and older one year before and two years after the 2008 USPSTF recommendation against testing in men aged 75 and older. The hypothesis was that overall PSA testing rates would drop from 2007 to 2010, similar to other studies,[7], [11], [12] and that variations in PSA testing rates would also decrease among PCPs.

## Methods

The overall approach was to identify PCPs who cared for at least 20 men aged 75 and older in both 2007 and 2010, and examine the percent of each PCP’s patients who underwent PSA testing in those years. Given the variation among PCPs,[10] 20 patients are sufficient to provide testing estimates with a reliability of >0.85 [19].

### Ethics Statement

The UTMB institutional review board approved this retrospective study. Patient and provider information were de-identified in the Medicare claims data. Informed consent was not obtained because patient and provider information were de-identified in the Medicare claims data.

### Identification of Patients and Primary Care Physicians

Using 100% Texas Medicare claims data for 2004–10, two cohorts of men were identified. The first included men aged 75 years or older as of 1/1/2007; residing in Texas in 2007; with no claims related to prostate cancer in 2004–06 (International Classification of Diseases, Ninth Revision, Clinical Modification [ICD-9-CM] diagnosis codes: 185, V104.6, 222.2, 233.4, 236.5; ICD-9-CM procedure codes: 60.21, 60.29, 60.3–60.6; and Current Procedure Terminology [CPT] codes: 55801, 55810, 55812, 55815, 55821, 55842, 55845); and with continuous Medicare Parts A and B without health maintenance organization (HMO) coverage during 2004–07. [20], [21] The second cohort, selected using the same criteria, included men aged 75 years or older as of 1/1/2010. Information on each man’s demographics, Medicare coverage and HMO enrollment was obtained from the Medicare enrollment files. Men were then selected from both cohorts with an identifiable PCP. A man’s PCP was identified by the method of Shah et al. [22] as the physician who saw that man on two or more occasions in an outpatient setting for evaluation and management (CPT codes 99201–99205 and 99211–99215) in 2007 or 2010 and had a Health Care Financing Administration (HCFA) specialty code in family medicine, general practice, internal medicine or geriatrics. Physicians were identified by the National Provider Identifier (NPI). If a man had more than one identified physician, the one who provided the most evaluation and management services was assigned as his PCP. In the case of ties, the most recently visited physician was assigned as the PCP. The sample was then restricted to men with an identifiable PCP who had at least 20 such patients in both 2007 and 2010. The final study cohorts contained 37,264 men in 2007 and 45,692 men in 2010, cared for by 1,083 PCPs.

### Patient Characteristics

Men were categorized by age (75–79, 80–84 and 85+ years). Comorbidity was assessed by the Elixhauser comorbidity measure based on Medicare Carrier files (claims for physician services), Outpatient Statistical Analysis Files (claims for hospital outpatient services) and Medicare Provider Analysis and Review files (claims for inpatient stays) in 2006 or 2009. The number of comorbidities was categorized as none, 1, 2, 3 or≥4. [20], [21] Race/ethnicity, obtained from the Medicare Part D denominator file, was categorized as White, Black, Hispanic/Latino, or Other/Unknown. Medicaid eligibility (yes or no) was used as a proxy for poverty and was measured by state buy-in fields in the Medicare enrollment file. The patient’s county of residence was categorized into urban, non-urban and rural, according to definitions developed by the US Department of Agriculture. [23] The percentage of high school graduates in the patient’s zip code area was obtained from the US Census data.

### PCP characteristics

PCP age and gender in 2007 and board certification in 2007 and 2010 were obtained from the American Medical Association (AMA) Masterfile, which were linked with Medicare claims via the provider NPI. A PCP’s specialty in 2007 was obtained from the HCFA specialty field in the carrier files and was categorized as Family Medicine (including family medicine and general practice) or Internal Medicine (including general internal medicine and geriatrics).

### PSA Testing

Each man in the 2007 and 2010 cohorts was assessed for claims for any PSA testing (Carrier files with CPT codes of 84153 and Healthcare Common Procedural Coding Systems Code of G0103) in 2007 and 2010, respectively. Both PSA testing ordered by any physician and that ordered by a man’s own PCP were identified, but for most analyses the outcome was PSA testing ordered by the PCP. Men were excluded who had any diagnoses in the three months prior to PSA test that suggested symptoms associated with prostate cancer (e.g., hematuria, weight loss, urinary obstruction), because such diagnoses or symptoms suggest that the PSA was obtained as a diagnostic test and not a screening test [8], [9].

### Statistical Analysis

In 2013, descriptive analysis was used to summarize the patient and PCP characteristics and PSA testing rates in 2007 and 2010 stratified by these characteristics. Multilevel logistic regression modeling was done separately for the 2007 and 2010 cohorts to (1) evaluate variation among PCPs using intra-class correlation (ICC) statistics; (2) evaluate associations between PCP characteristics and PSA testing, adjusting for patient characteristics; (3) estimate PSA testing rates for each PCP, adjusting for patient characteristics and within-PCP clustering; and (4) identify PCPs with a significantly lower or higher than average PSA testing rate. The 1,083 PCPs were then ranked based on their adjusted PSA testing rates, from lowest to highest, for both 2007 and 2010. The ICCs for PCPs from the 2007 and 2010 multilevel models were compared using Levine’s test for equal variance. Finally, a model was constructed including both the 2007 and 2010 cohorts, and tested for interactions between year (2007 or 2010) and PCP characteristics. SAS version 9.2 (SAS Institute, Cary, NC) was used for all statistical analyses.

## Results

In all, 1,083 PCPs had >20 men aged 75+ in their patient panels in both 2007 (total men = 37,264) and 2010 (total men = 45,692). Table 1 presents the percent of patients who underwent PSA screening in 2007 and 2010, stratified by characteristics of patients and their PCPs, and also the odds ratios for receiving PSA screening adjusted for all characteristics in a multivariable multilevel model. The 2007 rate of PSA screening ordered by the patient’s PCP (33.2%) decreased to 30.6% in 2010 (p<0.001). The rates of all PSA screening by any physician were 45.2% in 2007 and 42.4% in 2010 (p<0.001). In both 2007 and 2010, the odds of PSA testing declined for patients with increasing age or greater comorbidities. Patient race/ethnicity and socioeconomic status had little effect. PCP characteristics independently associated with higher odds of PSA testing included a greater number of men aged 75 years or older in their patient panels, and Internal Medicine specialty.

**Table 1:** Patient and primary care physician characteristics and their associations with prostate specific antigen (PSA) screening.

Patient Characteristics*	Number of patients (% receiving PSA screening ordered by PCP)		OR (95% CI)	
	2007	2010	2007	2010
**Overall**	37,264 (33.2)	45,692 (30.6)	−	−
**Age (years)**				
75–79	17,487 (39.1)	15,728 (37.8)	1.00	1.00
80–84	11,682 (31.8)	15,654 (31.6)	0.76 (0.72, 0.80)	0.75 (0.71, 0.79)
85+	8,095 (22.6)	14,310 (21.6)	0.42 (0.40, 0.45)	0.42 (0.39, 0.44)
**Race/Ethnicity**				
White	31,348 (34.0)	38,037 (31.0)	1.00	1.00
Black	905 (30.5)	1,165 (30.6)	0.96 (0.81, 1.14)	1.01 (0.87, 1.17)
Hispanic	4,603 (28.4)	5,922 (28.1)	0.99 (0.89, 1.11)	1.08 (0.97, 1.19)
Other	382 (34.0)	548 (29.9)	1.10 (0.84, 1.45)	0.83 (0.65, 1.06)
**Numbers of comorbidities**				
0	5,591 (35.2)	5,936 (33.9)	1.00	1.00
1	11,323 (37.2)	12,758 (34.8)	1.11 (1.03, 1.20)	1.07 (0.99, 1.15)
2	9,191 (33.5)	11,611 (31.5)	0.92 (0.85, 1.00)	0.90 (0.83, 0.98)
3	5,297 (31.6)	6,973 (27.8)	0.83 (0.76, 0.91)	0.74 (0.68, 0.81)
4+	5,462 (23.9)	8,414 (22.9)	0.56 (0.51, 0.62)	0.57 (0.52, 0.62)
**Medicaid eligibility**				
No	33,719 (33.9)	41,477 (31.0)	1.00	1.00
Yes	3,545 (27.0)	4,215 (27.1)	0.84 (0.75, 0.94)	0.93 (0.84, 1.04)
**Urban/Rural**				
Metro	28,882 (33.8)	35,104 (30.8)	1.00	1.00
Non-Metro	7,657 (31.3)	9,743 (29.7)	0.96 (0.88, 1.06)	1.07 (0.98, 1.17)
Rural	702 (30.5)	839 (30.3)	1.08 (0.87, 1.33)	1.13 (0.93, 1.39)
**Percent high school graduates in the zip code area**				
<75%	8,786 (30.8)	10,275 (28.4)	1.00	1.00
75–83%	8,642 (23.2)	10,757 (28.4)	0.96 (0.88, 1.04)	0.96 (0.88, 1.04)
84–90%	9,332 (25.0)	11,426 (30.9)	1.05 (0.96, 1.14)	1.03 (0.95, 1.12)
>90%	9,404 (25.2)	11,888 (34.1)	1.10 (1.01, 1.20)	1.06 (0.98, 1.15)
**PCP Characteristics**	**Number of PCPs (% of their patients receiving PSA ordered by PCP)**		**OR (95% CI)**	
	**2007**	**2010**	**2007**	**2010**
**Overall**	1,083 (33.2%)	1083 (30.6%)	−	−
**Age (years)**				
< = 50	432 (31.0%)	(29.6%)	1.00	1.00
50–60	438 (33.8%)	(30.4%)	1.03 (0.89, 1.19)	0.95 (0.81, 1.11)
>60	202 (36.5%)	(32.2%)	1.15 (0.95, 1.39)	1.03 (0.84, 1.27)
**Gender**				
Female	45 (26.3%)	45 (23.8%)	1.00	1.00
Male	1,027 (33.5%)	1,027 (30.6%)	1.31 (0.93, 1.84)	1.30 (0.89, 1.89)
**Number of Male Patients 75+ in 2007/2010 in their patient panel**				
20–25	345 (29.3%)	139 (24.7%)	1.00	1.00
26–35	365 (31.7%)	344 (28.8%)	1.10 (0.93, 1.30)	1.29 (1.01, 1.65)
36–50	243 (35.4%)	336 (35.4%)	1.35 (1.12, 1.62)	1.47 (1.15, 1.88)
>50	130 (36.2%)	264 (31.4%)	1.30 (1.04, 1.63)	1.36 (1.05, 1.76)
**Specialty**				
Family Medicine	442 (31.2%)	442 (28.9%)	1.00	1.00
Internal Medicine	641 (34.4%)	641 (31.6%)	1.13 (0.98, 1.30)	1.16 (1.00, 1.35)
**Board Certified in 2007/2010**				
Yes	828 (33.6%)	790 (30.5%)	1.00	1.00
No	70 (29.6%)	108 (29.0%)	0.92 (0.70, 1.22)	0.94 (0.74, 1.20)

*There are missing data for patient race/ethnicity (n = 26 in 2007 and n = 20 in 2010) urban/rural (n = 23 in 2007 and n = 6 in 2010) and education (n = 1100 in 2007 and n = 1344 in 2010); PCP characteristics are missing data on age (n = 11 in 2007 and 2010) and board certification (n = 188) in 2007 and 2010).

**Figure 1:**
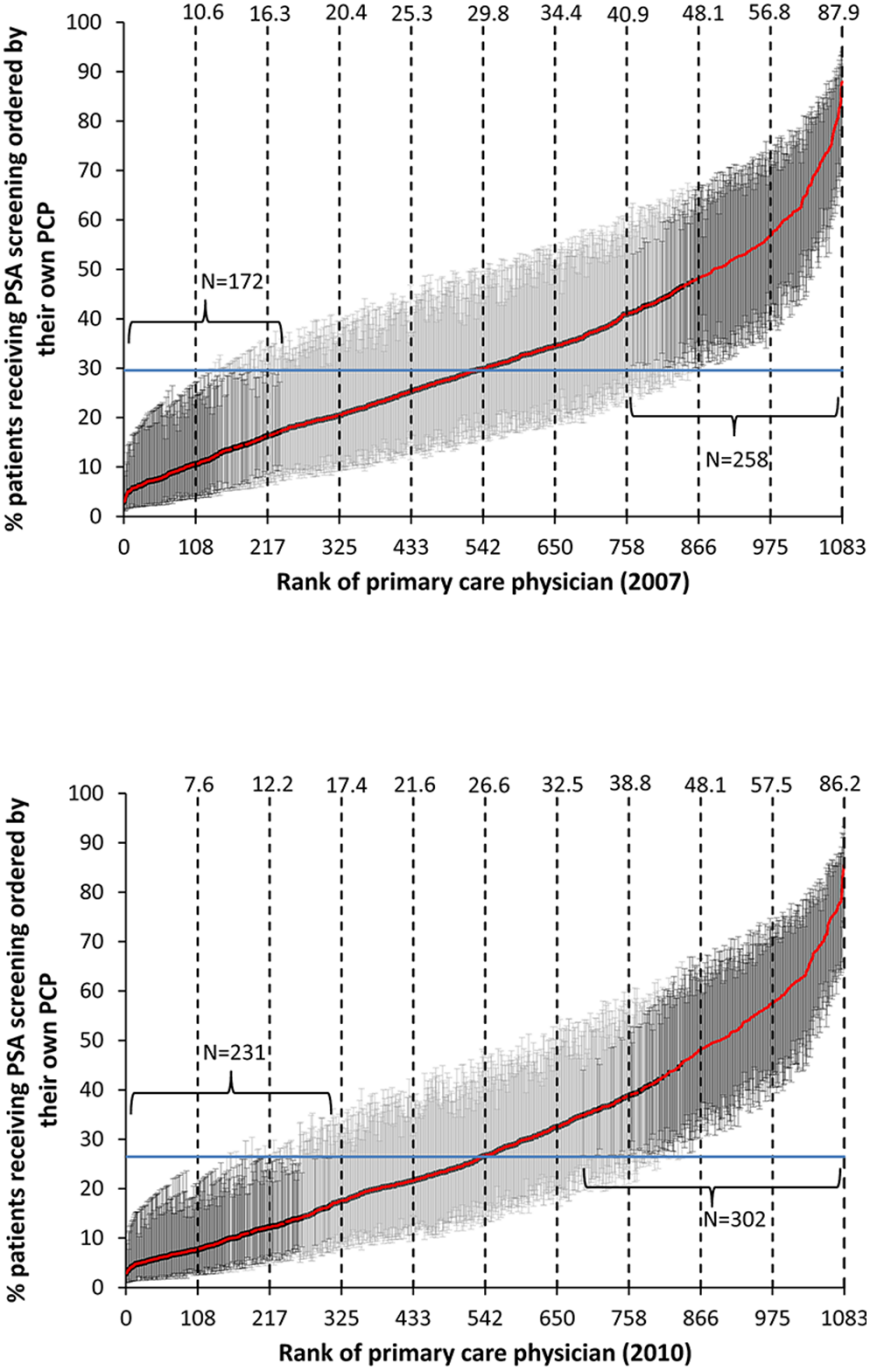
Cumulative distribution of 1,083 Texas primary care physicians (PCPs) by the adjusted percentage of their male patients age 75 and older who underwent prostate specific antigen (PSA) testing ordered by the PCP in 2007 (top panel) and 2010 (bottom panel). Only PCPs with at least 20 male patients 75+ in their panels in both years are included. The vertical lines denote the 95% confidence intervals of the estimates, derived from the multilevel models presented in Table 1. Dark lines indicate PCPs whose PSA testing rate was significantly different from the mean rate for all PCPs.

The models shown in Table 1 were then used to estimate PSA testing rates for each PCP in 2007 and 2010, adjusted for patient characteristics. Figure 1 presents cumulative distributions of adjusted PSA testing rates for each of the 1,083 PCPs in 2007 and 2010. PCPs varied substantially in testing rates in both years. PCPs with rates significantly higher or lower than the mean rate are indicated by bold lines. In 2007, 258 (24%) PCPs had rates significantly greater than the mean, with an average rate of 57.5%, while 172 (16%) PCPs had significantly lower rates, with an average rate of 9.7%. For 2010, the number of PCPs significantly higher and lower than the mean increased (p<0.001), with 302 (28%) PCPs with significantly higher rates (average rate 55.0%) and 231 (21%) with significantly lower rates (average rate 8.5%).

Also shown in Figure 1 are the rates by decile of PCP rank. For example, PCPs in the lowest decile in 2007 had rates <10.6%, with an average rate for those PCPs of 7.7%. In 2010 the lowest decile was <7.6%, with an average rate of 6.0%. In contrast, the cut points and average rates for the top decile of PCPs actually increased slightly between 2007 and 2010.

**Figure 2:**
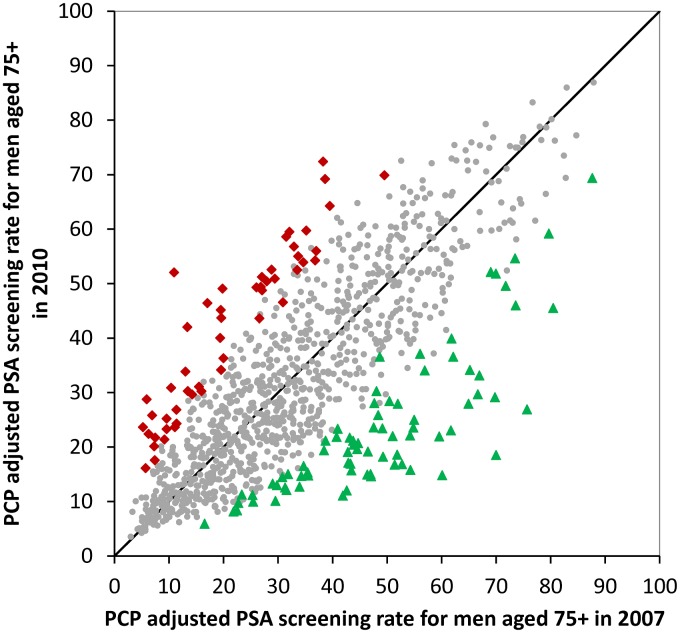
Scatterplot of the adjusted prostate specific antigen (PSA) screening rates in men 75+ for 1,083 PCPs in 2007 vs. 2010. The 51 PCPs with significantly higher rates in 2010 are indicated with red, while the 77 PCPs with significantly lower rates are in green. The results were generated from a multilevel model adjusting for patient characteristics and including both the 2007 and 2010 data in the same model.

The multilevel models presented in Table 1 also show the ICC at the PCP level for each year. In 2007 the ICC was 0.23; in 2010, it increased to 0.26 (p<0.001). This is consistent with the cumulative distributions shown in Figure 2, showing greater dispersion in PSA testing rates of PCPs in 2010 versus 2007. In both years, specific patient characteristics (age, comorbidity, education, etc.) explained less than 4% of the variance in receipt of PSA screening.

There was good stability in the PCP ranking between 2007 and 2010, with a Pearson correlation co-efficient of 0.63. Of the 216 PCPs in the top quintile of screening rates in 2007, 74.1% were in the first (49.1%) or second (25.0%) quintile in 2010. Similarly, of the 258 PCPs in Figure 1 with PSA screening rates significantly higher than the mean rate in 2007, 178 (69.0%) had significantly higher rates in 2010. Figure 2 graphs the adjusted PSA screening rates for each of the 1,083 PCPs in 2007 vs. 2010. It also shows the PCPs whose screening rates significantly increased (n = 51, indicated in red) or decreased (n = 77, indicated in green).

To determine if specific PCP characteristics were associated with changes in PSA screening rates by PCPs from 2007 to 2010, a multilevel multivariable model was constructed combining both years of data and including year (2010 vs. 2007) as a variable. After controlling for patient and PCP characteristics, the odds of receiving a PSA test from one’s PCP decreased 9% between 2007 and 2010 (OR = 0.91, 95% CI = 0.87; 0.96). This model showed significant interactions between year (2010 vs. 2007) and PCP age, board certification, and size of the PCP panel. As shown in Table 2, patients of PCPs older than age 60 experienced a 9% lower odds of receiving a PSA test in 2010 compared to 2007, versus a 4% increase for patients whose PCP was younger than age 50. Patients of PCPs with board certification experienced a 12% decrease in odds of PSA testing versus a 2% increase for patients of PCPs without board certification. Patients with PCPs with a high volume of older men in their panels also experienced a drop in odds of undergoing PSA screening.

**Table 2:** PCP characteristics associated with change in PSA screening rates from 2007 to 2010.

PCP Characteristics	OR (95% CI) of receivingPSA screening, 2010 vs. 2007
**Age (years)**	
< = 50	1.04 (0.96, 1.12)
51–60	0.97 (0.90, 1.04)
>60	0.91 (0.83, 0.99)
**Number of Patients 75+**	
20–25	1.03 (0.95, 1.12)
26–35	0.97 (0.90, 1.05)
36–60	1.00 (0.92, 1.08)
>50	0.88 (0.80, 0.96)
**Board Certified**	
Yes	0.88 (0.84, 0.92)
No	1.02 (0.89, 1.18)

PCP: primary care physician; PSA: prostate specific antigen.

## Discussion

In the 1970’s, Wennberg and colleagues described geographic variations in the receipt of surgical procedures. [17] The variation was much higher for operations with less clear-cut indications (such as tonsillectomy) than for those with clear indications (such as appendectomy). Wennberg et al. termed this phenomenon “preference sensitive care.” More recently, these investigators have expanded this concept to medical testing. [18], [24] Using clinical vignettes of patient scenarios, they found little variation in diagnostic and treatment decisions when the supporting evidence was clear cut, but considerable variation in situations with poor evidence. [18] With PSA testing, variation among PCPs actually increased after publication of the USPSTF recommendations, suggesting that consensus statements and guidelines did not reduce uncertainty.

The variation in PSA testing among PCPs is striking. PCPs in the lowest decile of testing differed nine- to ten-fold in PSA rates vs. those in the top decile. No other behaviors appear to have this high level of variation among PCPs. [19], [25]–[29] For example, previous reports show ICCs at the provider level of 0.10 and 0.09 for receipt of mammography[25] and colorectal cancer screening,[26] respectively, compared to the ICC of 0.26 for PSA screening in 2010.

PSA testing rates by PCPs overall decreased, from 33.2% in 2007 to 30.6% in 2010. However, as shown in Figure 1, PSA testing rates among physicians in the upper decile of PSA testing actually increased. The overall decrease in rates was driven by increases in the number of PCPs with lower rates. The cut points for the bottom seven deciles of PCPs in 2010 are all lower than in 2007. The result is a significant increase in variability among physicians, and an increase in the amount of overall variability in PSA testing attributable to the PCP.

The decline in PSA testing rates was greater in board certified and older PCPs. Several studies have found board certification associated with better adherence to guidelines,[30], [31] but those same studies tend to find that younger, more recently trained physicians are more adherent to guidelines. Patients of older PCPs (vs. younger PCPs) had higher odds of testing in 2007, but this association disappeared by 2010.

Investigators using interviews of PCPs have also documented considerable variation in PSA screening behavior. [7] Two groups surveyed PCPs after the release of the 2011 draft USPSTF recommendations against PSA screening in men of any age. [32], [33] PCPs varied considerably in whether they agreed with the recommendations and whether their PSA screening behavior would change as a result. Patient attitudes and preferences also clearly contribute to overtesting. For example, Schwartz et al.,[34] in a national telephone survey in 2001–02, found that 73% of males disagreed that they would ever stop getting PSA screening; 77% said they would try to continue the test even if their physician recommended against it. In a qualitative study, Torke et al. [35] found that older adults view cancer screening as a moral obligation.

Another contributing factor to the variation among providers in PSA screening may be the lack of complete consensus in the recommendations on PSA screening provided by various professional organizations. In general, organizations representing primary care and/or preventive medicine have recommended against PSA screening, while oncology and urology organizations have a broader spectrum of views. The US Preventive Services Task Force and primary care organizations such as the American College of Physicians and the American Academy of Family Practice recommend against PSA screening at any age. [3], [36], [37] The American Cancer Society and the American Urological Association recommend a patient-centered individualized approach to decisions, but discourage testing in older men. [4], [5] The Large Urology Group Practice Association and some other urology groups are more favorable to PSA screening, though they still discourage screening in men with less than 10 years life expectancy. [38], [39] All of the organizations generate press releases and have public websites to disseminate their diverse and conflicting recommendation, which presumably contributes to the lack of clarity among PCPs and their older male patients.

However, even given these differences in recommendations, there may be more consensus than is realized about PSA screening in older men. Even the most pro-screening groups do not recommend it for men with less than 10 years life expectancy. Using a validated algorithm to predict life expectancy using a man’s age and degree of comorbidity, [40] it is not possible to define a cohort of men aged 80 or older with a life expectancy of greater than 10 years. Only 12% of 80 year old men survive for 10 years, and it is not possible to prospectively identify them. Nevertheless, we found >30% such men received PSA screening in 2007 and 2010.

Previous reports have documented overuse of PSA screening in older men. Walter and colleagues [9] studied PSA testing in 2003 of men aged 70 and older cared for at US Veteran’s Affairs (VA) facilities, and found little influence of health status on testing rates. For example, with men aged 85 years and older, 34% in the best health and 36% in the worst health had PSA tests. Bynum et al. [8] analyzed 2003 Medicare data and found an overall PSA screening rate of 17.2% for men aged 80 years and older, with variation in testing rates across regions from 2% to 38%. The current data, from 2007 and 2010, suggest no improvement in these patterns. Ross et al. [11] compared PSA testing rates for men 75 and older in the months immediately before and after change in USPSTF recommendations, using the 5% Medicare non cancer sample from the Surveillance Epidemiology and End Results (SEER) Tumor Registry, representing 28% of the US population. They noted a 2% absolute decrease in testing rates. Howard et al. [12] compared testing rates on approximately 2,400 men aged 75 and older in 2006–07 to 2200 in 2009–10 in the Medicare Current Beneficiary Survey and found a 5.3% absolute decrease in testing rates between 2006 and 2010. In contrast, self-reported PSA screening rates did not change between 2005 and 2010 in men aged 75 and older in the national Health Interview Survey. [41] Other reports have found little[42] or no[43] change in PSA testing rates in response to the publication of clinical trials. The overall picture, then, is of modest effects of published evidence and consensus recommendations on PSA screening, with substantial and increasing variation in screening behavior among PCPs. Part of the variation in PCP testing behavior likely reflects variations in the attitudes of their patient panels about such testing. Perhaps the increasing variations among PCPs reflect differences in willingness or ability to confront the issue with patients who equate screening with good medical care.

A major implication of these findings relates to targeting interventions to discourage PSA testing, particularly of men with limited life expectancy. The very high variability among PCPs suggests that PCPs would be an excellent target for intervention efforts. For example, overtesting rates have been suggested as quality measures of PCPs. [44], [45] Other approaches could include eliminating reimbursement for screening PSA tests in, for example, men aged 80 and older. Medicare recently decided to continue reimbursement for PSA screening with no age limitation [46].

The study has limitations. First, while we excluded patients with a history of prostate cancer, and those with recent symptoms suggestive of prostate cancer, this method undoubtedly did not eliminate all cases where the PSA testing was in response to symptoms, and not true screening. However, it is not plausible that the prevalence of such symptoms would vary greatly among men cared for by different PCPs. Second, our sample was restricted to men in fee-for-service Medicare. Evidence suggests that screening rates in HMOs and in the VA system are lower. [9], [47] Third, the study was limited to Texas. PSA screening rates are somewhat higher in southern states than in other regions. [8] Finally, an ongoing concern about physician profiling is reliability, which reflects an estimate of how much of measured variability is due to real differences in behavior. [19], [48] For example, Adams et al. [48] showed that most measures of physician-level resource utilization for specific episodes of care had reliabilities of less than 0.70, indicating poor reliability. Because of the high variation among PCPs in PSA testing, these estimates of PSA testing rates had a reliability of >0.85 for PCPs with 20 or more patients in their panel. All PCPs assessed had at least 20 male patients aged 75+ in their panels.

In conclusion, the continued high levels of PSA testing in older men, and the high variation among PCPs in rates of PSA testing, suggest that interventions at the PCP level may be useful.
